# The Effects of Positive Allosteric Modulators of α7–nAChR on Social Play Behavior in Adolescent Rats Prenatally Exposed to Valproic Acid

**DOI:** 10.3390/ph15111417

**Published:** 2022-11-16

**Authors:** Kinga Gzielo, Agnieszka Nikiforuk

**Affiliations:** Maj Institute of Pharmacology Polish Academy of Sciences, Department of Behavioral Neuroscience and Drug Development, 12 Smętna Street, 31-343 Kraków, Poland

**Keywords:** autism, valproic acid, alpha7–nicotinic receptor, positive allosteric modulator, social play, ultrasonic vocalization, communication, animal models, rats

## Abstract

There is still no effective treatment that addresses the core symptoms of autism spectrum disorders (ASD), including social and communication deficits. A comprehensive body of evidence points to the cholinergic system, including alpha7–nicotinic acetylcholine receptors (α7–nAChRs), as a potential target of pharmacotherapy. A promising approach is based on positive allosteric modulators (PAMs) of these receptors due to their advantages over direct agonists. Nevertheless, α7 n–AChR ligands have not been widely studied in the context of autism. Therefore, using one of the most widely used rodent models of ASD, that is, prenatal exposure to valproic acid (VPA), we examined the impact of α7–nAChR PAMs (PNU–120596 and CCMI) on socio-communicative behavior during social play in adolescent male and female rats. The current study demonstrated that PAM treatment affected certain aspects of socio-communicative behavior in adolescent rats. Accordingly, PNU–120596 ameliorated deficient play abilities in VPA-exposed males, as revealed by increased play time during a social encounter. In addition, this compound enhanced the emission of ultrasonic vocalizations that accompanied playful interactions. Moreover, we observed the overall effect of PNU–120596 on non-playful forms of social behavior (i.e., social exploration) and acoustic parameters (i.e., the duration) of emitted calls. The present results suggest the ability of α7–nAChR PAMs to facilitate socio-communicative behavior in adolescent rats.

## 1. Introduction

Autism spectrum disorders (ASD) are now a serious public health concern because of their early onset, lifelong persistence and high levels of associated impairment. However, despite the urgent need, there is still no effective treatment for the core symptoms of ASD. Therefore, several targets have gained attention as possible new treatment options. For example, the cholinergic abnormalities found in autistic patients and ASD animal models, combined with the clinical and preclinical efficacies of acetylcholinesterase inhibitors (AChEIs), point to the cholinergic system as a target for the pharmacotherapy of this disorder [1]. In particular, a growing body of evidence supports the role of the alpha7–nicotinic acetylcholine receptor (α7–nAChR) subtype in ASD pathogenesis and suggests its potential utility as a therapeutic target [1]. Nevertheless, the strategies based on α7–nAChR ligands have not been widely studied (but see also [2,3,4]).

Promising therapeutic intervention to activate α7–nAChRs is the possibility of using positive allosteric modulators (PAMs) that have been proposed as an alternative strategy to that based on orthosteric agonists [5]. A general difference between PAMs and agonists is that PAMs do not activate the receptor directly but instead function to potentiate responses to activation by agonists, thereby preserving the temporal integrity of neurotransmission. Consequently, α7–nAChR PAMs may exert beneficial activity without generating side effects like those mediated by orthosteric agonists, especially after long-term treatment. Based on their functional properties, α7–nAChR PAMs are generally divided into type I (e.g., CCMI, also known as AVL–3288) and type II (e.g., PNU–120596). Type I PAMs potentiate the agonist-induced peak current but do not affect the desensitization processes. On the other hand, the action of type II PAMs is accompanied by profound retardation of the kinetics of desensitization. This class of compounds has recently gained attention as a potential candidate in preclinical and early clinical drug development [1,6].

A first step in identifying possible drug targets for ASD symptomatology requires insight from animal models of this disorder. One of the most widely used rodent models of ASD is prenatal exposure to valproic acid (VPA) [7]. Behavioral abnormalities observed in VPA-exposed animals correspond to the core symptoms defining ASD, i.e., impaired communication/social interactions and stereotypic/repetitive behaviors. Moreover, this neurodevelopmental model is considered a useful tool for testing therapeutic interventions for ASD [8].

Social and communication deficits, a main autistic feature, can be successfully modeled in laboratory conditions. In particular, rats are very social animals that exhibit highly developed and complex social behavior patterns, and social play (also called the rough-and-tumble play) behavior in adolescent rats is one of the earliest forms of social interaction [9]. The fact that social play deficits are widely described in autistic children and have a key role in identifying and diagnosing ASD supports the translational value of preclinical assessment of social play behavior.

Playing rats also communicate using ultrasonic vocalizations (USVs) at frequencies of about 50 kHz that differ in their durations and patterns of frequency modulations [9]. While rats’ ultrasonic vocalizations cannot be directly compared to human communication, quantitative and qualitative changes in USV emission may serve as a readout of communicative deficits in laboratory conditions [10,11,12]. Consequently, previous studies demonstrated that prenatal VPA exposure induced social play deficits accompanied by changes in USV emission [13]. Thus, adolescent rat play is an early behavioral biomarker of neurodevelopmental changes and, as such, may be used for testing novel drug targets.

Therefore, the current study aimed to test the ability of α7–nAChR PAMs, PNU–120596 and CCMI to reverse VPA-induced socio-communicative deficits observed during social play in adolescent rats.

## 2. Results

### 2.1. Social Play Behavior

**Figure 1:**
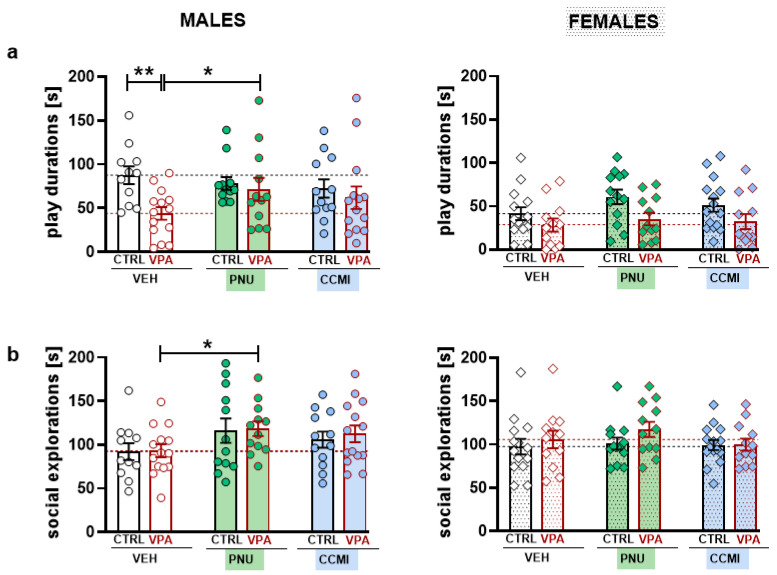
The effect of α7–nAChR PAMs on social play behavior (**a**) and social exploration (**b**) in VPA and CTRL male and female rats. The tested compounds (or vehicle, VEH) were administered at a dose of 1 mg/kg 30 min prior to testing. Data are presented as a mean ± SEM of the time spent by a pair of rats on social play (**a**) and social explorations (**b**). The results were expressed as the summed score of both animals in a pair. Symbols: ** *p* < 0.01, * *p* < 0.05, planned comparisons.

VPA exposure significantly decreased the time rats spent engaged in social play (*p* = 0.0008, Tukey post hoc test following a significant VPA treatment effect: F [1,141] = 13.68, *p* = 0.0003, Figure 1a). However, between-treatment comparisons within each sex group revealed a significant play reduction only in VEH–VPA males (t = 3.289, *p* = 0.001, planned comparisons). Moreover, PNU–120596 (but not CCMI) administration significantly increased social play in VPA males compared to the vehicle-treated VPA group (t = 2.107, *p* = 0.0369, planned comparisons, Figure 1a). Consequently, VPA males treated with PNU–120596 did not differ from their vehicle-exposed counterparts (i.e., the PNU–CTRL group, Figure 1a). On the contrary, PAM treatment did not facilitate play behavior in VPA females (Figure 1a).

Moreover, PNU–120596 treatment significantly increased non-playful forms of social interaction, i.e., social exploration (*p* = 0.034, Tukey post hoc test following a significant PAM treatment effect: F [1,141] = 3.172, *p* = 0.045, Figure 1b). Comparisons within sex groups revealed that the significant PNU–120596 effect was demonstrated only in VPA males (t = 2.013, *p* = 0.046), and a trend towards increment was observed in CTRL males (t = 1.804, *p* = 0.073, planned comparisons, Figure 1b). In contrast to PNU–120596 action, CCMI did not significantly affect social exploration.

### 2.2. Ultrasonic Vocalizations

**Figure 2:**
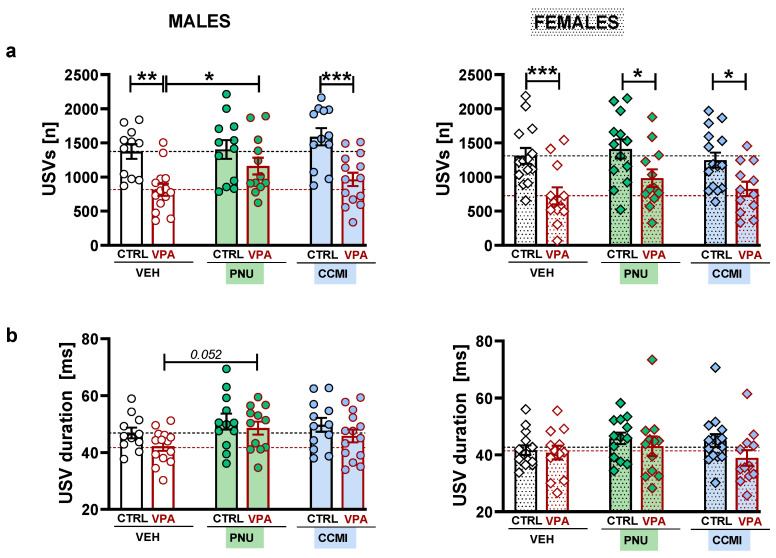
The effect of α7–nAChR PAMs on USV emission in VPA and CTRL male and female rats. Data are presented as a mean ± SEM of the number of 50-kHz USVs (**a**) and the average durations of the emitted calls (**b**). The tested compounds (or vehicle, VEH) were administered at a dose of 1 mg/kg 30 min prior to testing. Symbols: *** *p* < 0.001, ** *p* < 0.01, * *p* < 0.05, planned comparisons.

VPA exposure also decreased USV emission during social play (*p* < 0.0001, Tukey post hoc test following a significant VPA treatment effect: F [1,141] = 48.91, *p* < 0.0001, Figure 2a). Comparisons within each sex group revealed that a significantly lower number of USVs was observed in both VEH–VPA males (t = 3.297, *p* = 0.001) and females (t = 3.544, *p* = 0.0005, planned comparisons, Figure 2a).

Moreover, the number of USVs emitted by PNU–VPA males was significantly higher than in the VEH–VPA male group (t = 2.091, *p* = 0.038, planned comparisons, Figure 2a). VPA males treated with PNU–120596 also did not differ from the PNU–CTRL males (Figure 1a). On the other hand, CCMI did not significantly increase the USV number in VPA males, and the CCMI–VPA group was still significantly different from the CCMI–CTRL group (t = 3.782, *p* = 0.0002, planned comparisons, Figure 2a).

However, PAM treatment did not affect USV emission in VPA females. Therefore, the USV number in PNU–VPA and CCMI–VPA females did not differ from that of the VEH–VPA female group and were significantly lower than in their CTRL counterparts (t = 2.537, *p* = 0.012 and t = 2.599, *p* = 0.0103, for PNU–120596 and CCMI groups, respectively, planned comparisons, Figure 2a).

The analysis of acoustic parameters of calls revealed that USVs emitted by VPA rats were overall shorter (*p* = 0.018, Tukey HSD post hoc test following a significant VPA treatment effect: F [1,141] = 6.457, *p* = 0.0121, Figure 2b) and of a narrower bandwidth (*p* = 0.0167, Tukey HSD post hoc test following a significant VPA treatment effect: F [1,141] = 6.242, *p* = 0.0136, Figure S1) as compared to the control groups. Moreover, PNU–120596 (but not CCMI) administration increased the durations of emitted calls compared to the VEH-treated animals (*p* = 0.0167, Tukey HSD post hoc test following a significant PAM treatment effect: F [2,141] = 3.155, *p* = 0.045, Figure 2b). Planned comparisons showed a trend toward an increased duration in PNU–VPA males compared to VEH–VPA males (t = 1.956, *p* = 0.0524, planned comparisons, Figure 2b). PNU–120596 also tended to decrease the peak frequency of male calls; however, this effect was demonstrated only when the CTRL and VPA groups were calculated together (t = 1.813, *p* = 0.0718, planned comparisons, Figure S1). We did not find any other between-group differences in the measured acoustic parameters.

The analysis of distinct USVs categories revealed that described PAM effects were mostly derived from changes in the emission of the most represented call types, i.e., trills and one-component frequency-modulated calls (Figure S2). However, we did not find any effects of PAMs on the percentage distribution of call types (Figure S3).

## 3. Discussion

The current study demonstrated that PAM treatment might affect certain aspects of socio-communicative behavior in adolescent rats. Accordingly, PNU–120596 ameliorated deficient play abilities in VPA-exposed males, as revealed by increased play time and USV emission. Moreover, we observed the overall effect of PNU–120596 on social exploration and the duration of emitted calls.

In line with previous studies [13], prenatal VPA exposure reduced the level of social play behavior. Likewise, the significantly shortened play time was demonstrated only in males. However, due to sexual dimorphism in juvenile play, females were generally less engaged in playful interactions than males, which may create a floor effect, thereby masking potential deficits. Nevertheless, the effects of PNU–120596 on play behavior and non-playful forms of interactions (i.e., social exploration) were evident only in VPA males. This action does not seem to be attributed to unspecific locomotor side effects, as rats’ open-field activity was not affected by PAM treatments (Figure S4).

To our knowledge, α7–nAChR PAMs or direct agonists have not been evaluated in the juvenile rat model of autistic-like social play disturbances. There is also limited data on the effectiveness of other cholinergic strategies in this regard. Nevertheless, most of the available data comes from mouse studies using an easily quantifiable sociability measure reflected as a tendency to spend more time with a conspecific than an inanimate object. Accordingly, the AChEI donepezil improved the sociability of VPA-exposed mice [14]. Donepezil was also effective against sociability impairment in the BTBR mouse model of idiopathic autism [15], and a similar effect was demonstrated for nicotine [16]. The specific role of the α7–nAChR was suggested in the study by Yoshimura et al. [2], demonstrating that CCMI increased social approach in BTBR mice, and the α7–nAChR-selective antagonist, methyllycaconitine, blocked this effect. Moreover, the beneficial effect of curcumin on social deficits in BTBR mice was mediated through α7–nAChRs [4]. Finally, the α7–nAChR agonist rescued social deficits in a Rett syndrome model, the Mecp2 (Methyl–CpG-binding Protein 2) knockout mice [3]. Although there is little data on the effectiveness of selective α7–nAChR ligands in ASD models, this strategy has also been proven effective against schizophrenia-like disturbances, including social withdrawal. For example, CCMI and PNU–120596 ameliorated social interaction deficits in rat models of schizophrenia based on pharmacological blockade of the NMDA receptor [17,18]. The current study corroborates and extends this data demonstrating that this strategy may also be effective against early ASD-relevant social impairments.

In line with the previous study, deficient play abilities in VPA-exposed males were accompanied by reduced USV emission. Although VPA females also exhibited vocalization deficits, PNU–120596-induced USV increases were observed only in males. While we are unaware of any studies examining the impact of α7–nAChR ligands on USVs, nicotine did not significantly affect the number of 50-kHz calls recorded from a single animal suggesting a lack of the drug’s rewarding properties per se [19]. Therefore, we can speculate that the elevated USV production was triggered mainly by a social context and corresponded to increased play behavior. It may also explain the limited effectiveness of the tested compounds in VPA females, which lacked changes on a behavioral level.

The administration of α7–nAChR PAMs also affected the acoustic feature of emitted USVs. Most notably, we observed the overall effect of PNU–120596 on the call’s duration. In addition, this compound also tended to decrease the peak frequencies of male calls. As the decreased duration and elevated peak frequencies may represent atypical USV features in VPA animals [13], the demonstrated tendency of PNU-120596 to ameliorate these parameters may contribute to its prosocial actions.

The current study suggested that PNU–120596 was more effective in promoting social behavior than CCMI. However, it cannot be excluded that a higher dose of CCMI than 1 mg/kg would be required to elicit an adequate response. For example, Yoshimura et al. [2] demonstrated that CCMI at a dose of 3 mg/kg increased the social approach in BTBR mice. Nevertheless, our previous study demonstrated that both compounds were comparably effective in the social interaction test in rats when administered at 1 mg/kg. Similarly, in the Unal et al. [17] study, CCMI at 1 mg/kg reduced rats’ avoidance behavior during a social encounter. Alternatively, the distinct properties of PNU–120596 as a type II PAM, including an ability to prolong agonist responses due to delayed desensitization and/or reactivation of desensitized receptors, might be responsible for its advantageous behavioral outcome.

## 4. Materials and Methods

### 4.1. Animals

Pregnant dams (Sprague–Dawley rats, N = 24) were obtained from Charles River (Sulzfeld, Germany) on gestation day (GD) 9–10. They were housed individually in polycarbonate cages: 26.5 (width) × 18 (height) × 42 (length) cm. On postnatal day (PND) 21, pups were weaned and separated by sex and litter into groups of 3–5 rats. Females and males were housed in different temperature-controlled (21 ± 1 °C) and humidity-controlled (40–50%) colony rooms under a 12/12 h light/dark cycle (lights on at 06:00 h). Food and water were available ad libitum. Behavioral testing was performed during the light phase of the light/dark cycle. The experiments were conducted in accordance with the European Guidelines for animal welfare (2010/63/EU) and were approved by the II Local Ethics Committee for Animal Experiments at the Maj Institute of Pharmacology, Polish Academy of Science, Krakow, Poland.

### 4.2. VPA Administration

The pregnant dams were injected intraperitoneally (i.p.) with VPA at a dose of 500 mg/kg (N = 12) or vehicle (N = 12) on GD 12.5. VPA (Sigma–Aldrich, Poznan, Poland) was dissolved in physiological saline. VPA and physiological saline (vehicle) were administered at a volume of 2 mL/kg. The dose and time of administration of VPA were based on previous reports demonstrating autistic-like disturbances, including social and communicative deficits (e.g., [7,13]). In line with previous reports [13], prenatal exposure to VPA did not produce toxic signs in the dams or the offspring. The detailed litter characteristic is provided in Figure S5. In total, 71 males and 87 females were born from 12 vehicle-treated dams and 88 males and 75 females from 12 poly VPA-treated dams.

### 4.3. Drug Administration

CCMI (Tocris, Bristol, UK) and PNU–120596 (Ascent Scientific, Bristol, UK) were dissolved in an aqueous 10% Cremophor solution (vehicle). The tested compounds and vehicle (VEH) were injected intraperitoneally (i.p.) at a volume of 2 mL/kg of body weight 30 min before testing. The tested dose (1 mg/kg) was based on our previously reported studies demonstrating drug-evoked prosocial effects [18]. To minimize the risk of a litter effect, offspring were randomly distributed across the testing condition (i.e., VEH, CCMI, or PNU–120596).

### 4.4. Social Play Test (30–35 PND)

The test procedure was conducted between 30–35 PND in same-sex, same-treatment pairs, as previously described [13]. One day before the test, the rats were transported to the experimental room and weighed; the backsides of one-half of the animals were marked with a Pentel permanent marker. Next, they were individually adapted to the test area for about 5 min. The dimly illuminated (15 Lux) test area consisted of a rectangular, polycarbonate cage (width × height × length: 38 × 20 × 59 cm) with approximately 2 cm of wood shavings covering the floor. On the testing day, each rat was isolated in a non-transparent plastic cage (width × height × length: 22 × 15 × 28 cm) for 2.5 h before the test. Then two unfamiliar (various cages/litters) rats of matched body weights (±5 g) were placed in the test area, and their behaviors were recorded for 10 min using the Observer software (Noldus Information Technology, The Netherlands).

The behavior of each rat in a pair was separately analyzed by an experienced observer blind to the experimental conditions using the Observer software. The results were expressed as the summed score of both animals in a pair. The duration of scored social play included pouncing (one of the rats attempts to rub the nape of the conspecific’s neck) and pinning (upon contact with the nape, the recipient animal fully rotates to a supine position while the other subject stands over it) behaviors, considered the main indices of social play behavior in rats. In addition, the time rats spent chasing, wrestling and boxing was also included in the play duration.

Moreover, we also measured the time of non-playful forms of social interaction (mostly sniffing/anogenital sniffing, but also grooming and climbing behaviors), regarded as social exploration.

The numbers of pairs in control (CTRL) and VPA groups used in the analysis were: N = 11 (VEH–CTRL males), N = 12 (PNU–CTRL males), N = 12 (CCMI–CTRL males), N = 14 (VEH–VPA males), N = 12 (PNU–VPA males), N = 14 (CCMI–VPA males), N = 14 (VEH–CTRL females), N = 13 (PNU–CTRL females), N = 15 (CCMI–CTRL females), N = 12 (VEH–VPA females), N = 12 (PNU–VPA females), N = 12 (CCMI–VPA females).

### 4.5. USV Recording

As previously described [13], the rats’ vocalizations were recorded during the entire test session (i.e., 10 min) using a frequency response range of 2 kHz–200 kHz microphone (UltraSoundGate Condensor Microphone CM16/CMPA, Avisoft Bioacoustics, Berlin, Germany) suspended 25 cm above the floor of the test area. Microphone signals were fed into an UltraSoundGate 416H (Avisoft Bioacoustics, Berlin, Germany) before the analog signal was digitized with a sampling rate of 200 kHz and a 16-bit resolution. Acoustic data were recorded using Raven Pro: Interactive Sound Analysis Software, version 1.5 (The Cornell Lab of Ornithology Bioacoustics Research Program, Ithaca, NY, USA). The calls were manually marked on the computer screen and counted by an experienced user, blind to the treatment, using the Raven Pro software. The spectrograms were generated with a fast Fourier transform (FFT)-length of 512 points and a time-window overlap of 75% (100% frame, Hamming window).

We analyzed: (a) the number of 50-kHz USVs (expressed as a total number of USVs emitted by a pair of rats) and the following features of 50-kHz USV: (a) the call duration (length of the call, measured in milliseconds), (b) the bandwidth (the difference between the highest and lowest frequencies, a measure of frequency modulation, expressed in kHz) and (c) the peak frequency (the frequency in kHz at which maximal energy occurs within the selection). Moreover, we manually divided the calls (based on their acoustic call features) into the following general types: short calls, flat calls with a near-constant frequency and frequency-modulated calls. The frequency-modulated calls were subsequently classified as trills, one-component (complex, ramp and inverted-U) and multi-component (multi-step, step-up, step-down and composite) calls.

### 4.6. Statistics

Data were analyzed by three-way ANOVAs with the VPA treatment (CTRL and VPA), PAM treatment (VEH, PNU–120596 and CCMI) and sex (male and female) as the between-subject factors. Data on the percentage distribution of call categories were arcsine-transformed and subjected to ANOVA analysis with call type as an additional factor. Detailed ANOVA results are presented in Table S1 (Supplementary materials).

When there was a significant main effect of the VPA model or PAM treatment, we used the Tukey HSD post hoc tests to assess overall between-group differences. In addition, the planned comparisons of Least Squares means were used to compare treatment conditions within a given sex. The normality of data distribution was evaluated by the Kolmogorov–Smirnov test. The effect size was estimated using partial eta squared (ŋp2). Statistical significance was set at *p* < 0.05. The statistical analyses were performed using Statistica 12.0 for Windows.

## 5. Conclusions

The present results may suggest the therapeutic potential of α7–nAChR PAMs to facilitate adolescent socio-communicative behavior.

## Data Availability

Data are contained within the article and Supplementary Materials.

## Supplementary Materials

The following supporting information can be downloaded at: https://www.mdpi.com/article/10.3390/ph15111417/s1, Figure S1. Acoustic characteristics of emitted 50-kHz calls, Figure S2. Call categories, Figure S3. Percent distribution of USVs within categories, Figure S4. Exploratory activity, Figure S5. Litter characteristics, Table S1. ANOVA results.
