# Supplementary material for: The Effects of Positive Allosteric Modulators of α7–nAChR on Social Play Behavior in Adolescent Rats Prenatally Exposed to Valproic Acid
Source: Pharmaceuticals (Basel). 2022 Nov 16;15(11):1417. doi: 10.3390/ph15111417 (PMC9697996; doi:10.3390/ph15111417)
Supplement: Supplementary file 1 [file pharmaceuticals-15-01417-s001.zip › pharmaceuticals-1993219-supplementary.pdf]

Figure S1. Acoustic characteristics of emitted 50-kHz calls: bandwidth (a) and the peak frequency (b).

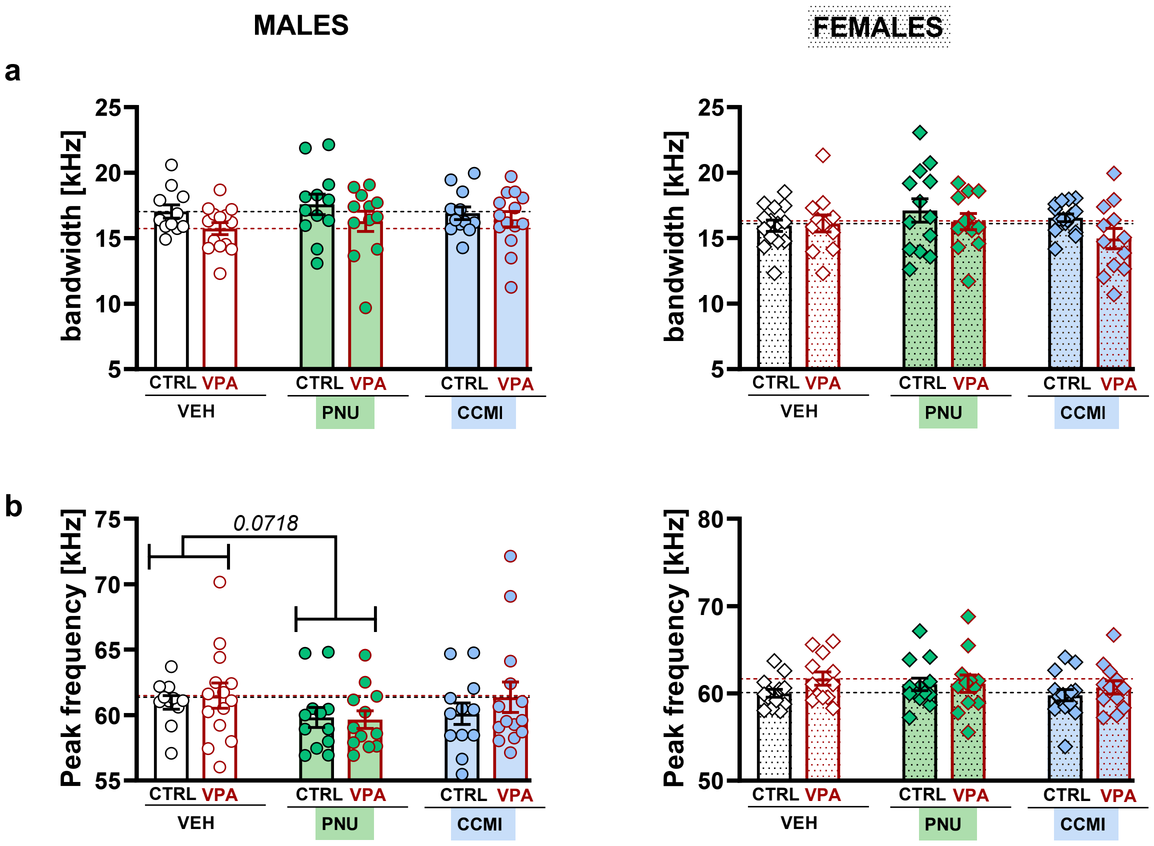

Data are presented as a mean ± SEM of bandwidth (a) and the peak frequency (b) of emitted calls.

Figure S2. Call categories.

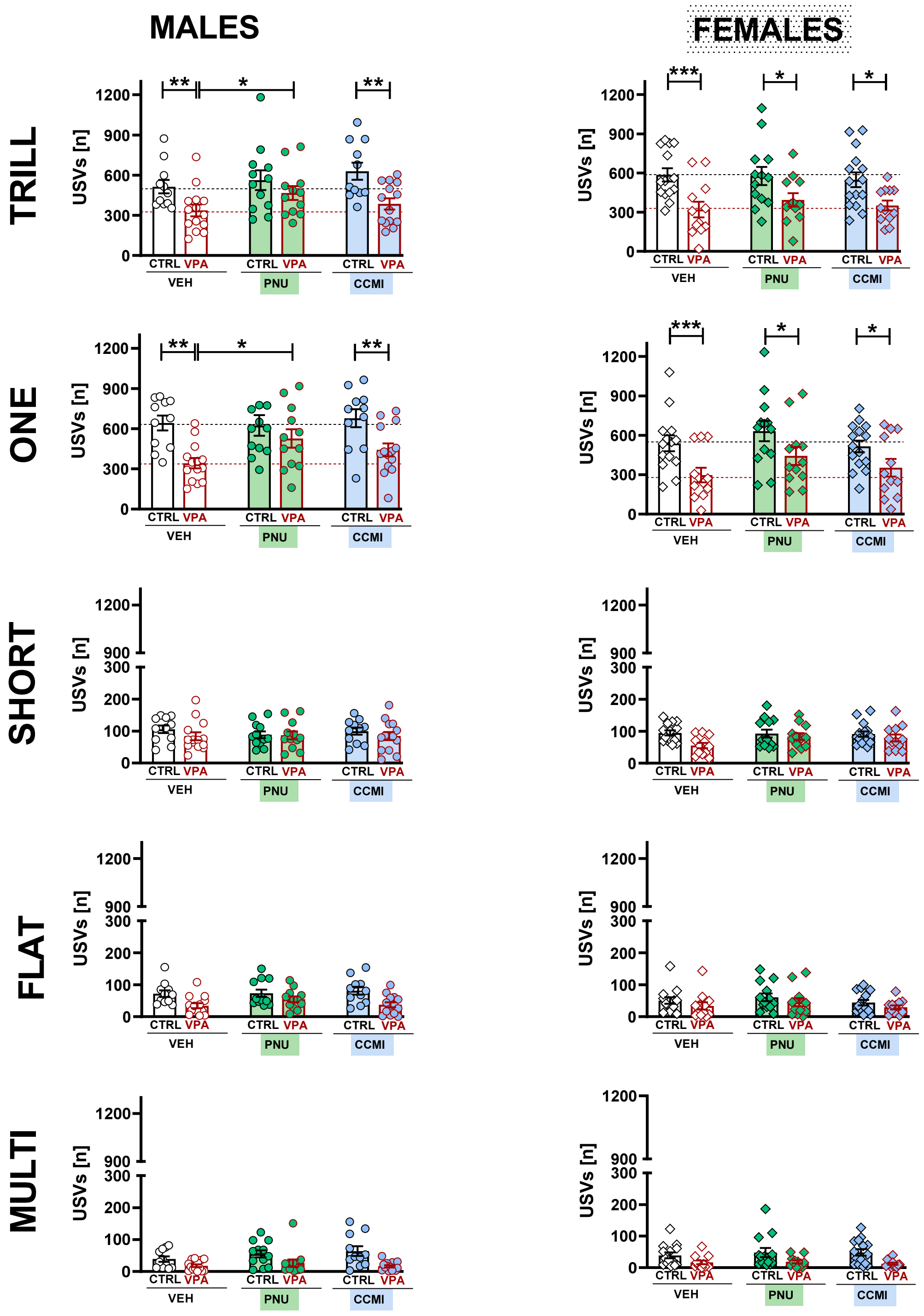

Data are presented as a mean ± SEM of the number of a given call type. Symbols:***p<0.001, **p<0.01, *p<0.05 (planned comparisons).

Figure S3. Percent distribution of USVs within categories.

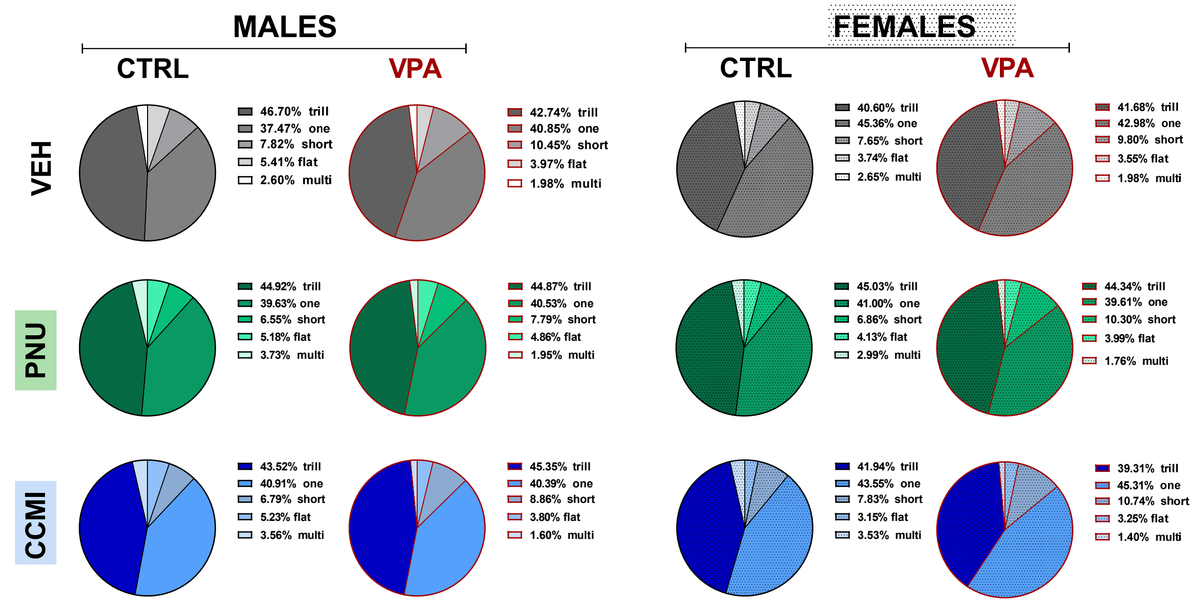

Data are presented as the percentage of calls within each category.

Figure S4. Exploratory activity.

PAM treatment did not affect the distance traveled by control rats in the open field (insignificant PAM treatment effect or PAM x sex interaction). However, open-field activity was higher in females than males (p=0.029, Tukey HSD post hoc test following a significant sex effect: F[1,25]=5.87, p=0.023).

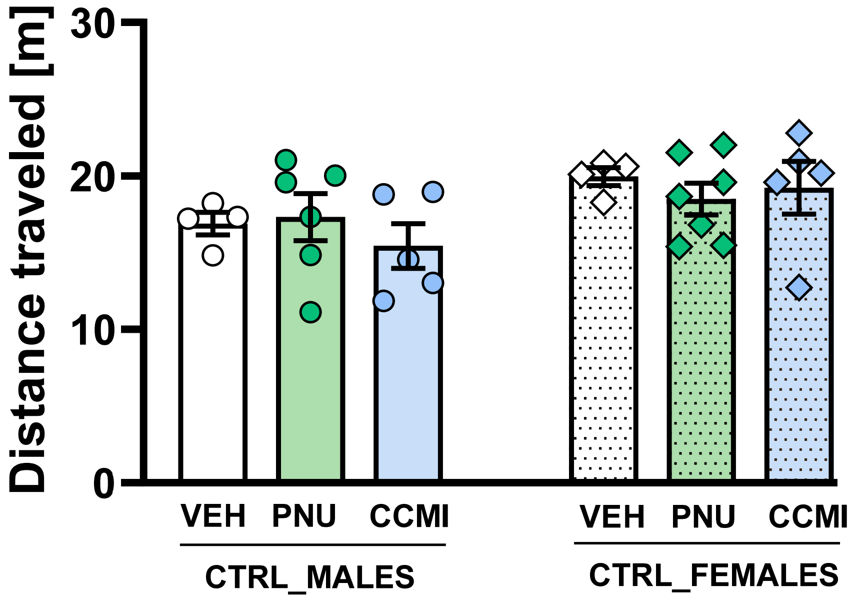

Data are presented as a mean ± SEM of the distance traveled by rats in the open field.

Table 1.S6. ANOVA results.

EFFECT	Degr. of freedom	F	p	Partial eta-squared	Observed power
sex	1,25	5,870	0,02298	0,1901	0,6439
PAM treatment	2,25	0,293	0,74869	0,0229	0,0913
PAM*sex	2,25	0,582	0,56609	0,0445	0,1358

Figure S5. Litter characteristics

In total, 71 males and 87 females were born from 12 vehicle-treated dams and 88 males and 75 females from 12 VPA-treated dams. VPA did not affect gestation length. There were no significant effects of VPA exposure on litter size (average number ≈13.6), but a trend toward a VPA x sex interaction (male/female ratio for VEH/VPA animals ≈ 0.76/1.16).

**Mean number of pups (12 CTRL litters and 12 VPA litters)**		
	**CTRL**	**VPA**
Male	5.9	7.3
Female	7.7	6.3
total	13.6	13.6

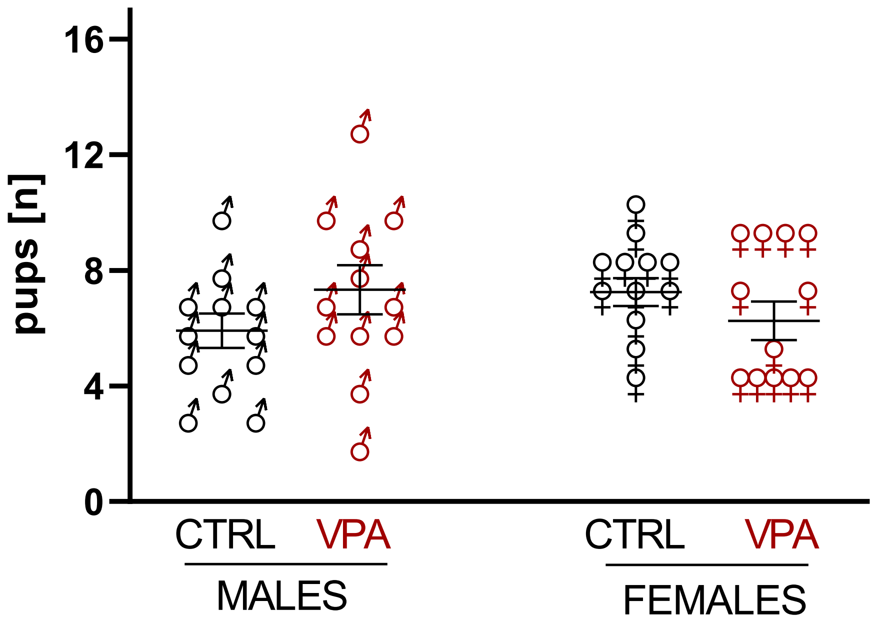

Data are presented as a mean ± SEM of the number of pups in each litter.

**ANOVA results.**					
EFFECT	**Degr. of freedom**	**F**	**p**	**Partial eta-squared**	**Observed power**
VPA	1 ,44	0,100	0,75380	0,0023	0,0610
sex	1 ,44	0,036	0,85068	0,0008	0,0539
VPA*sex	1 ,44	3,351	0,07396	0,0708	0,4328

Table S1. ANOVA results.

Data were analyzed by three-way ANOVAs with the VPA treatment (CTRL and VPA), PAM treatment (VEH, PNU-120596, and CCMI), and sex (male and female) as the between-subject factors.

1. **Behavior during the social interaction test**

a. **Social play**

EFFECT	Degr. of freedom	F	p	Partial eta-squared	Observed power
VPA treatment	1,141	13,681	0,00031	0,0884	0,9567
PAM treatment	2,141	1,426	0,24372	0,0198	0,3016
sex	1,141	26,855	0,00000	0,1600	0,9993
*VPA*PAM*	2,141	0,664	0,51626	0,0093	0,1599
*VPA*sex*	1,141	0,011	0,91650	0,0001	0,0512
*PAM*sex*	2,141	0,103	0,90228	0,0015	0,0655
*VPA*PAM*sex*	2,141	1,939	0,14768	0,0268	0,3967

b. **Social exploration**

EFFECT	Degr. of freedom	F	p	Partial eta-squared	Observed power
VPA treatment	1,141	1,282	0,25942	0,0090	0,2028
PAM treatment	2,141	3,172	0,04492	0,0431	0,5999
sex	1,141	0,295	0,58793	0,0021	0,0839
*VPA*PAM*	2,141	0,115	0,89158	0,0016	0,0673
*VPA*sex*	1,141	0,238	0,62656	0,0017	0,0773
*PAM*sex*	2,141	1,328	0,26831	0,0185	0,2831
*VPA*PAM*sex*	2,141	0,337	0,71432	0,0048	0,1031

2. **Ultrasonic vocalizations during the social interaction test.**

a. **The number of 50 kHz calls**

EFFECT	Degr. of freedom	F	p	Partial eta-squared	Observed power
VPA treatment	1,141	48,914	0,00000	0,2576	1,0000
PAM treatment	2,141	2,408	0,09370	0,0330	0,4792
sex	1,141	3,916	0,04977	0,0270	0,5022
*VPA*PAM*	2,141	1,101	0,33530	0,0154	0,2405
*VPA*sex*	1,141	0,000	0,98359	0,0000	0,0500
*PAM*sex*	2,141	0,624	0,53741	0,0088	0,1527
*VPA*PAM*sex*	2,141	0,677	0,50992	0,0095	0,1621

b. **The acoustic characteristics of calls**

i) **Duration**

EFFECT	Degr. of freedom	F	p	Partial eta-squared	Observed power
VPA treatment	1,141	6,457	0,01213	0,0438	0,7136
PAM treatment	2,141	3,155	0,04567	0,0428	0,5973
sex	1,141	12,740	0,00049	0,0829	0,9435
*VPA*PAM*	2,141	0,349	0,70620	0,0049	0,1050
*VPA*sex*	1,141	0,020	0,88655	0,0001	0,0523
*PAM*sex*	2,141	0,317	0,72889	0,0045	0,0997
*VPA*PAM*sex*	2,141	0,443	0,64285	0,0062	0,1210

ii) **Bandwidth**

EFFECT	Degr. of freedom	F	p	Partial eta-squared	Observed power
VPA treatment	1,141	6,242	0,01363	0,0424	0,6989
PAM treatment	2,141	1,236	0,29380	0,0172	0,2657
sex	1,141	2,021	0,15731	0,0141	0,2923
*VPA*PAM*	2,141	0,207	0,81305	0,0029	0,0818
*VPA*sex*	1,141	0,129	0,71989	0,0009	0,0647
*PAM*sex*	2,141	0,339	0,71319	0,0048	0,1033
*VPA*PAM*sex*	2,141	1,147	0,32043	0,0160	0,2491

iii) **Peak Frequency**

EFFECT	Degr. of freedom	F	p	Partial eta-squared	Observed power
VPA treatment	1,141	2,303	0,13136	0,0161	0,3256
PAM treatment	2,141	0,732	0,48296	0,0103	0,1721
sex	1,141	0,110	0,74108	0,0008	0,0625
*VPA*PAM*	2,141	0,635	0,53118	0,0089	0,1548
*VPA*sex*	1,141	0,134	0,71458	0,0010	0,0653
*PAM*sex*	2,141	1,581	0,20932	0,0219	0,3307
*VPA*PAM*sex*	2,141	0,226	0,79818	0,0032	0,0848

3. Call categories

a. Number

EFFECT	Degr. of freedom	F	p	Partial eta-squared	Observed power
VPA	1,705	96,828	0,00000	0,12076	1,0000
PAM	1,705	4,767	0,00879	0,01334	0,7937
sex	2,705	7,752	0,00551	0,01088	0,7940
VPA*PAM	1,705	2,180	0,11381	0,00615	0,4463
VPA*sex	2,705	0,001	0,97688	0,00000	0,0501
PAM*sex	1,705	1,235	0,29154	0,00349	0,2696
VPA*PAM*sex	2,705	1,340	0,26261	0,00379	0,2897
call	2,705	481,581	0,00000	0,73207	1,0000
VPA*call	4,705	20,436	0,00000	0,10390	1,0000
PAM*call	4,705	1,330	0,22496	0,01487	0,6148
VPA*PAM*call	8,705	0,657	0,72948	0,00740	0,3084
sex*call	8,705	2,127	0,07582	0,01192	0,6320
VPA*sex*call	4,705	0,279	0,89133	0,00158	0,1123
PAM*sex*call	4,705	0,568	0,80416	0,00641	0,2666
VPA*PAM*sex*call	8,705	0,469	0,87820	0,00530	0,2212

b. Percentage distribution

EFFECT	Degr. of freedom	F	p	Partial eta-squared	Observed power
VPA	1,705	0,002	0,96420	0,00000	0,0502
PAM	1,705	0,002	0,99850	0,00000	0,0502
sex	2,705	0,007	0,93386	0,00001	0,0508
*VPA*PAM*	1,705	0,007	0,99258	0,00002	0,0511
*VPA*sex*	2,705	0,000	1,00000	0,00000	0,0500
*PAM*sex*	1,705	0,002	0,99793	0,00001	0,0503
*VPA*PAM*sex*	2,705	0,004	0,99577	0,00001	0,0506
call	2,705	1571,775	0,00000	0,89917	1,0000
*VPA*call*	4,705	1,907	0,10744	0,01071	0,5781
*PAM*call*	4,705	1,159	0,32140	0,01298	0,5433
*VPA*PAM*call*	8,705	0,102	0,99916	0,00116	0,0789
*sex*call*	8,705	4,545	0,00125	0,02514	0,9438
*VPA*sex*call*	4,705	0,288	0,88553	0,00163	0,1146
*PAM*sex*call*	4,705	0,995	0,43809	0,01117	0,4694
*VPA*PAM*sex*call*	8,705	0,919	0,50016	0,01032	0,4334
